# The blood-to-plasma ratio and predicted GABA_A_-binding affinity of designer benzodiazepines

**DOI:** 10.1007/s11419-022-00616-y

**Published:** 2022-03-16

**Authors:** Kieran R. Manchester, Laura Waters, Shozeb Haider, Peter D. Maskell

**Affiliations:** 1 School of Nursing and Healthcare Leadership, University of Bradford, Bradford, UK; 2 School of Applied Sciences, University of Huddersfield, Huddersfield, UK; 3 School of Pharmacy, University College London, London, UK; 4 Forensic Medicine and Science, University of Glasgow, Glasgow, UK

**Keywords:** QSAR, Designer benzodiazepines, New psychoactive substances, Blood-to-plasma ratio, GABA_A_ receptor

## Abstract

**Purpose:**

The number of benzodiazepines appearing as new psychoactive substances (NPS) is continually increasing. Information about the pharmacological parameters of these compounds is required to fully understand their potential effects and harms. One parameter that has yet to be described is the blood-to-plasma ratio. Knowledge of the pharmacodynamics of designer benzodiazepines is also important, and the use of quantitative structure–activity relationship (QSAR) modelling provides a fast and inexpensive method of predicting binding affinity to the GABA_A_ receptor.

**Methods:**

In this work, the blood-to-plasma ratios for six designer benzodiazepines (deschloroetizolam, diclazepam, etizolam, meclonazepam, phenazepam, and pyrazolam) were determined. A previously developed QSAR model was used to predict the binding affinity of nine designer benzodiazepines that have recently appeared.

**Results:**

Blood-to-plasma values ranged from 0.57 for phenazepam to 1.18 to pyrazolam. Four designer benzodiazepines appearing since 2017 (fluclotizolam, difludiazepam, flualprazolam, and clobromazolam) had predicted binding affinities to the GABA_A_ receptor that were greater than previously predicted binding affinities for other designer benzodiazepines.

**Conclusions:**

This work highlights the diverse nature of the designer benzodiazepines and adds to our understanding of their pharmacology. The greater predicted binding affinities are a potential indication of the increasing potency of designer benzodiazepines appearing on the illicit drugs market.

**Supplementary Information:**

The online version contains supplementary material available at 10.1007/s11419-022-00616-y.

## Introduction

Benzodiazepines are amongst the most commonly prescribed drugs in the world because of their sedative, anticonvulsant, and anxiolytic effects [1, 2]. Most benzodiazepines exert their anxiolytic and sedative actions by binding to γ-aminobutyric acid type A (GABA_A_) receptors [3]. The endogenous ligand for this receptor is γ-aminobutyric acid (GABA), the primary inhibitory neurotransmitter in the brain. When GABA binds to the GABA_A_ receptor on a neuron, it causes the cell to become hyperpolarised. In turn, this inhibits the transmission of an action potential. Benzodiazepines bind to an allosteric site on the GABA_A_ receptor and potentiate the response of the receptor to GABA, thereby further decreasing neuron excitability. The inhibition of action potentials decreases motor stimulation and cortical activity, which results in the physiological effects of benzodiazepines [4]. These pharmacological effects and the clinical properties of benzodiazepines also cause them to be misused [5, 6]. This misuse has increased significantly around the world since ~ 2007 when, to avoid relevant regional drug control legislation, there was a shift to the use of benzodiazepines that had been licensed for clinical use in other parts of the world but not in the country of use [7]. The first two benzodiazepines to be detected by the European Monitoring Centre for Drugs and Drug Addiction (EMCDDA) were nimetazepam and phenazepam, followed by etizolam in 2011 [7]. When these compounds were placed under international control, there was a move to orphan benzodiazepines, drugs that had been developed by drug companies but that had not progressed to market. As of 2021, the EMCDDA is monitoring 30 so-called “designer” or new psychoactive substance (NPS) benzodiazepines [8, 9].

Knowledge of the pharmacology of these designer benzodiazepines is important for harm reduction around the world as due to their illicit nature, they have not gone through the standard research and clinical testing of licensed medicines [10]. Although there has been a large amount of research into the physicochemical and pharmacological properties of benzodiazepines [11–13]. There are still gaps in our basic pharmacological knowledge, for example in the potential potency of emerging designer benzodiazepines and the blood-to-plasma ratio. Knowledge of the blood-to-plasma ratio is important to allow the comparison of designer benzodiazepine concentrations where plasma may have been analysed (such as in clinical laboratories) or when blood has been analysed [such as driving under the influence of drugs (DUID) cases]. Reliable interpretations from these analyses and any equivalences drawn requires knowledge of the blood-to-plasma ratio [14]. Difficulties in comparing the concentrations of designer benzodiazepines in blood and plasma have been described because of a lack of published values [15]. As well as finding utility in toxicological analyses, blood-to-plasma ratios have also been used to predict concentration–time profiles, compound clearance from plasma, and hepatic clearance, allowing further prediction of the pharmacology of designer benzodiazepines [16–18]. Potency evaluation of novel compounds can be carried out via functional studies or models such as quantitative structure–activity relationship (QSAR) modelling [19–21]. QSAR provides a quick and inexpensive method to gain an understanding of the pharmacodynamics of designer benzodiazepines. In this work, we have described the prediction of binding affinity (using QSAR) and the determination of the blood–plasma ratio of a selection of designer benzodiazepines, allowing a greater understanding of their pharmacology and potential harms to users.

## Materials and methods

### Chemicals, reagents, and biological samples

The test compounds (chlorpromazine, diazepam, nitrazepam, and quinine) were obtained from Sigma-Aldrich (Dorset, UK). The designer benzodiazepines (deschloroetizolam, diclazepam, etizolam, meclonazepam, phenazepam, and pyrazolam) were obtained from Chiron (Trondheim, Norway). All compounds were received as powdered solids. Dimethyl sulfoxide (DMSO) (LC–MS grade) and acetonitrile (LC–MS grade) were obtained from Fisher Scientific (Leicestershire, UK). Phosphate-buffered saline (PBS) tablets were obtained from Sigma-Aldrich (Dorset, UK).

Human blood (pooled, from three male donors and three female donors) was obtained from Seralab (West Sussex, UK). Blood was received chilled with sodium citrate as an anticoagulant and kept at 4 °C until use. The blood was used prior to its expiration date. Blood haematocrit was 41%, as provided by the supplier.

### Determination of the blood-to-plasma ratio

A method of determining the blood-to-plasma ratios of compounds has been well described in the literature [22]. In this method, only analysis of the plasma is required. Briefly, the compound is spiked into equal volumes of whole blood and plasma. Following equilibration, the whole blood is centrifuged to extract the plasma. The extracted plasma and the original plasma are analysed to obtain compound concentrations. The blood-to-plasma ratio can then be calculated from Eq. 1 (shown below).

In this work, aliquots of whole blood (5 mL) were centrifuged at 2500 rpm for 20 min to provide plasma. This was performed on the day of the experiments. The plasma was removed and stored at 4 °C until use (typically within 2 h).

The compounds were dissolved in DMSO or acetonitrile to produce 5 mM stock solutions. Stock solutions were diluted with PBS (pH 7.4) to produce working solutions (50 µM) on the day of experimentation. Stock solutions were stored for no longer than 1 week. Appropriate volumes of working solutions were added to blood or plasma to yield a final compound concentration of 5 µM (final solvent concentrations were 0.1%) [23]. The treated blood or plasma samples were incubated for 1 h at 37 °C. Following incubation, the blood sample was removed and centrifuged at 2500 rpm for 20 min and the plasma was extracted. The 250 µL of extracted plasma (from spiked whole blood) and 250 µL of spiked plasma had ice-cold acetonitrile added to precipitate proteins (4:1 ratio of acetonitrile-to-plasma). Both plasma samples were then centrifuged at 10,000 rpm for 20 min. The supernatants were collected and evaporated under a stream of nitrogen using a Biotage Turbovap LV Evaporator. The evaporated samples were reconstituted in 150 µL acetonitrile and analysed using gas chromatography–mass spectrometry (GC–MS).

Four test compounds (chlorpromazine, diazepam, nitrazepam, and quinine) were chosen to validate this approach as they spanned a range of blood-to-plasma ratios, from 0.51 to 0.59 for diazepam to 1.46–2.00 for quinine [24–29]. The blood-to-plasma ratio determination was performed for the four test compounds and the designer benzodiazepines.

### GC–MS analysis of plasma samples

An Agilent 7890B GC with a 7693 autosampler and a 5977A MSD was used with a HP-5 MS 5% phenyl/95% methylpolysiloxane-fused silica capillary column (30 m × 0.25 mm, thickness 0.25 µm). Inlet port temperature was 280 °C; transfer line temperature was 250 °C. The carrier gas was helium with a constant flow rate of 1.2 mL/min. The split less injection volume was 1 µL. The temperature program consisted of an initial temperature of 60 °C for 2 min followed by a 30 °C/min ramp to 280 °C and a 10 min hold at 280 °C. The total run time was 19.3 min. The MS was operated in scan mode with electron impact ionization and the electron energy was 70.0 eV. Source temperature was 230 °C and the quad temperature was 150 °C.

Qualitative data analysis was conducted using ChemStation version F.01.01.2317 to confirm the presence of the analytes using their respective *m*/*z* values for qualifier ions (Table S1). One quantifier ion was selected for quantification of the analyte (identified as underlined in Table S1).

### Validation

The method was validated in terms of linearity, limit of quantitation (LOQ), limit of detection (LOD), accuracy, and precision according to ICH guidelines [30]. Data analysis was performed on Microsoft Excel. The validation methodology and results can be found in the Supplementary material.

### Calculation of blood-to-plasma ratio

The blood-to-plasma partition coefficient was calculated using Eq. 1

1 $${K}_{e/p}=\frac{1}{H}\times \left(\frac{{C}_{P\mathrm{Ref}}}{{C}_{P}}-1\right)+1,$$

where *K*_*e/p*_ is the red blood cell partition coefficient, *H* is the haematocrit, *C*_*PRef*_ is the concentration (µM) in the reference plasma, and *C*_*P*_ is the concentration (µM) in the plasma separated from the whole blood [22].

*K*_*e/p*_ describes the ratio of the concentration of drug in the red blood cells (not including plasma) to the concentration of drug in plasma. *K*_*e/p*_ can be converted to the blood-to-plasma ratio (*K*_*b/p*_) with Eq. 2

2 $${K}_{b/p}={(K}_{e/p}\times H)+\left(1-H\right),$$

where *K*_*b/p*_ is the blood-to-plasma ratio, *K*_*e/p*_ is the blood-to-plasma partition coefficient, and *H* is the haematocrit. *K*_*b/p*_ therefore describes the ratio of the concentration of drug in whole blood (containing both red blood cells and plasma) to the concentration of the drug in plasma [31].

### QSAR

A quantitative structure–activity relationship (QSAR) model has previously been used to predict the binding affinities to GABA_A_ receptors for 22 designer benzodiazepines [19]. This QSAR model was created from the structure of characterised benzodiazepines and GABA_A_ receptor binding, expressed as the logarithm of the reciprocal of concentration (log 1/c), where c is the molar inhibitory concentration (IC_50_) required to displace 50% of [3H]-diazepam from rat cerebral cortex synaptosomal preparations [32]. In this work, the same QSAR model was applied to nine designer benzodiazepines reported to the EMCDDA, plus gidazepam and its metabolite, desalkylgidazepam [9]. Briefly, 88 benzodiazepines were selected for building the QSAR model in Molsoft ICM-Pro [33]. These were converted from SMILES to 3D structures based on Merck Molecular Force Field (MMFF) atom type and force field optimisation. These compounds were then aligned by common substructure and confirmation to Ro 05-3061. Subsequently, the aligned compounds were clustered by Atomic Property Fields (APF) to identify benzodiazepines with poor alignment. At this point, 10 benzodiazepines with poor alignment were removed to improve model accuracy. From the remaining 78 aligned compounds, 9 compounds were selected using a random number generator based on atmospheric noise. These compounds were removed from the training set and used for final model validation. The residual 69 compounds were used as the training set to build a 3D QSAR model. The APF method, designed by MolSoft, uses the assignment of a 3D pharmacophore potential on a continuously distributed grid using physiochemical properties of the selected compound(s) to classify or superimpose compounds. These properties include: hydrogen-bond donors, acceptors, Sp2 hybridisation, lipophilicity, size, electropositivity/negativity, and charge [34]. Benzodiazepines were clustered by APF clustering and subjected to re-alignment using APF-based flexible superimposition to check if there was any outlier in the alignment. The compounds were used as the training set to build a 3D QSAR model. The APF 3D QSAR method was used where, for each of the 69 aligned compounds, the seven physicochemical properties were calculated and pooled together. Based on the binding activity data obtained from literature and the 3D aligned structures for the known benzodiazepines, weighted contributions for each APF component were obtained to allow quantitative activity predictions for the designer benzodiazepines. The optimal weight distributions were assigned by partial least-squares (PLS) methodology, where the optimal number of latent vectors for PLS was established by leave-one-out cross-validation on the training set. Then, the weighted contributions were added together. The designer benzodiazepines were assigned predicted binding values by calculating their fit within the combined QSAR APF. Any designer benzodiazepines were subjected to the conversion and alignment protocol before predicted binding data was obtained. Further details of the QSAR model used can be found in previously published work [19].

## Results

### Test compounds

The literature blood-to-plasma ratios for the test compounds, and the experimental blood-to-plasma ratios for the test compounds are displayed in Table 1. Chlorpromazine had an experimental blood-to-plasma ratio of 1.43 versus a literature range of 1.17–1.56. Diazepam had an experimental blood-to-plasma ratio of 0.59 versus a literature range of 0.51–0.59. Nitrazepam had an experimental blood-to-plasma ratio of 0.63 versus a literature range of 0.57–1.00. Quinine had an experimental blood-to-plasma ratio of 1.66 versus a literature range of 1.46–2.00.

**Table 1:** Experimental and literature (where available) blood-to-plasma ratios for all compounds in this work

Compound	*K*_*b/p*_ literature	*K*_*b/p*_ experimental	References
Chlorpromazine	1.17–1.56	1.43 ± 0.32	[23, 28, 29]
Diazepam	0.51–0.59	0.59 ± 0.02	[24–27]
Nitrazepam	0.57–1.00	0.63 ± 0.02	[23, 27, 36]
Quinine	1.46–2.00	1.66 ± 0.52	[18, 23]
Deschloroetizolam	N/A	0.68 ± 0.06	N/A
Diclazepam	N/A	0.82 ± 0.05	N/A
Etizolam	N/A	0.70 ± 0.03	N/A
Meclonazepam	N/A	0.83 ± 0.08	N/A
Phenazepam	N/A	0.57 ± 0.13	N/A
Pyrazolam	N/A	1.18 ± 0.03	N/A

### Designer benzodiazepines

The experimental blood-to-plasma ratios for designer benzodiazepines are displayed in Table 1. The lowest blood-to-plasma ratio was 0.57 for phenazepam. Deschloroetizolam had the next lowest blood-to-plasma ratio of 0.68, closely followed by etizolam with 0.70. Diclazepam and meclonazepam had blood-to-plasma ratios of 0.82 and 0.83, respectively. The highest blood-to-plasma ratio was 1.18 for pyrazolam.

### Binding affinity

The predicted binding affinities to GABA_A_ receptors for the designer benzodiazepines, expressed as log 1/c, are displayed in Table 2. Full structures of all the designer benzodiazepines are given in the Supplementary material, Tables S3-S7. Difludiazepam had the highest predicted binding affinity for the GABA_A_ receptor of the 1,4-benzodiazepines in this study of 9.16.

**Table 2:** Predicted binding affinities to the GABA_A_ receptor (log 1/c) for nine designer benzodiazepines plus gidazepam and its metabolite desalkylgidazepam

Name	Log 1/c predicted
Bentazepam	6.8769
Cinazepam	7.11038
Clobromazolam	10.14
Desalkylgidazepam	7.97322
Difludiazepam	9.16362
Flualprazolam	10.1289
Fluclotizolam	8.90603
Gidazepam	8.3262
Norfludiazepam	8.85335
Thionordazepam	7.08873
Tofisopam	5.02924

The two triazolobenzodiazepines clobromazolam and flualprazolam had predicted binding affinities of 10.14 and 10.13, respectively. The thienotriazolodiazepine fluclotizolam had a predicted binding affinity of 8.91. The thienodiazepine bentazepam had a predicted binding affinity of 6.88. The 2,3-benzodiazpine tofisopam had a predicted binding affinity of 5.03.

## Discussion

### Blood-to-plasma ratio of the test compounds

The four test compounds were chosen, because they spanned a range of blood-to-plasma ratios, from 0.51 to 0.59 for diazepam to 1.46–2.00 for quinine [24–29]. There is a large variation in reported blood-to-plasma ratios for many compounds in the literature. When blood-to-plasma ratios are not known, they are often assumed to be equal to 1.0 or equal to the blood-to-plasma ratios for other animals [23, 35].

The blood-to-plasma ratio of diazepam is commonly reported as 0.51–0.59 [24–27]. The experimental blood-to-plasma ratio in this work was 0.59 ± 0.02 which is similar to the values quoted in the literature of 0.51–0.59 [24–27]. Nitrazepam is commonly reported as having a blood-to-plasma ratio of 1.0 [36]. The origin of this value is unclear and could be from the common assumption that the blood-to-plasma ratio is equal to 1.0. In this work, an experimental blood-to-plasma ratio of 0.63 ± 0.02 was determined. One study in the literature quantitated benzodiazepines by 125-I radioimmunoassay and provided concentrations of nitrazepam in whole blood and plasma [27]. These were converted into a blood-to-plasma ratio of 0.57 ± 0.27 (*n* = 3), which is similar to the experimental value calculated in this work. Calculations from the same source report a blood-to-plasma ratio of 0.51 ± 0.10 for diazepam which is also similar to the value derived in this work. Chlorpromazine had an experimental blood-to-plasma ratio of 1.43 ± 0.32 in this work compared to a literature range of 1.17–1.56 [23, 28, 29]. Quinine had an experimental blood-to-plasma ratio of 1.66 ± 0.52 in this work compared to a literature range of 1.46–2.00 [18, 23].

The coefficients of determination for the GC–MS were lower than expected for some compounds (Table S2). However, all four test compounds had calculated blood-to-plasma ratios that were within the literature ranges. This allowed for determination of the blood-to-plasma ratios for the designer benzodiazepines in this work.

### Blood-to-plasma ratio of the designer compounds

The measured blood-to-plasma ratios for the six NPS-benzodiazepines ranged from 0.57 for phenazepam to 1.18 for pyrazolam.

The blood-to-plasma ratio of 0.57 ± 0.13 for phenazepam indicates extensive partitioning into the plasma and a low association with red blood cells. Phenazepam has a plasma protein binding of 98.3% which limits the amount that can bind to red blood cells [13]. Similarly, low blood-to-plasma ratios have been reported for benzodiazepines in the literature such as 0.51–0.59 for diazepam (0.59 reported in this work) [24–27].

Deschloroetizolam and etizolam had similar blood-to-plasma ratios of 0.68 ± 0.06 and 0.70 ± 0.03, respectively. A low blood-to-plasma ratio could be expected because of the relatively high plasma protein binding of these compounds (87.2% for Deschloroetizolam and 92.8% for etizolam) [13].

Meclonazepam had a blood-to-plasma ratio of 0.83 ± 0.08. Meclonazepam is structurally similar to clonazepam, differing only with the addition of a methyl group on the R_3_ position. Clonazepam is reported to have a lower blood-to-plasma ratio of 0.65 [37].

Diclazepam had a blood-to-plasma ratio of 0.82 ± 0.05. Diclazepam is structurally similar to diazepam, differing only with the addition of a chlorine atom at the 2’ position. Diclazepam has a reduced plasma protein binding (93.8%) compared to diazepam (99.0%) [13]. Therefore, a higher blood-to-plasma ratio for diclazepam can be expected (0.82) versus that of diazepam (0.59) on account of its lower plasma protein binding. However, it is important to note that plasma protein binding is not the only determinant of blood-to-plasma ratio, which involves many factors such as binding site, lipophilicity, molecular size, and chirality [31].

Pyrazolam had a blood-to-plasma ratio of 1.18 ± 0.03. This value indicates a greater association with red blood cells than the other benzodiazepines. This is not surprising as pyrazolam has been reported to have a plasma protein-binding value of 78.7% which is low amongst the benzodiazepines [13].

The lowest blood-to-plasma ratio that could be found in the literature for a benzodiazepine was 0.49 for temazepam (calculated from whole blood and plasma concentrations) [27]. A blood-to-plasma ratio of 0.62 has also been reported for temazepam [37]. The highest blood-to-plasma ratio that found be found in the literature was 1.14 for lorazepam [38]. A blood-to-plasma ratio of 0.60 has also been reported for lorazepam [16]. These large ranges highlight the uncertainty around the exact values and the need for accurate determination.

The data presented here highlight the wide variation in blood-to-plasma ratios for designer benzodiazepines, from 0.57 for phenazepam to 1.18 for pyrazolam. As a result of the differences in blood-to-plasma ratio, relying on the assumption that the value is often equal to 1.0 may be an unsuitable approach for designer benzodiazepines. The determination of blood-to-plasma ratios for designer benzodiazepines will assist in interpreting blood concentrations of designer benzodiazepines [15]. Although the main use of blood-to-plasma ratios has been the interpretation of toxicological analyses, the ratios have been also used to predict concentration–time profiles and clearance [16, 17].

Blood-to-plasma ratios for a range of illicitly used compounds have been published, including for 3,4-methylenedioxymethamphetamine (MDMA), Δ^9^-tetrahydrocannabinol (THC), γ-hydroxybutyric acid (GHB), phencyclidine (PCP), zopiclone, and opiates such as morphine, oxycodone, and fentanyl [39–44]. The data presented in this work add to our understanding of the blood-to-plasma ratios of compounds used as new psychoactive substances.

### Binding affinity

The predicted binding affinity to the GABA_A_ receptor for a range of designer benzodiazepines has previously been reported [19]. This work focused on nine new benzodiazepines reported to the EMCDDA since 2017. The predicted binding affinity was also calculated for gidazepam, and its metabolite, desalkylgidazepam. Gidazepam is a prescription drug in Russia and Ukraine, and as it is not under international control, it may appear as a designer benzodiazepine in the future [9].

A large range of predicted binding affinities was observed in this work, from 5.03 for tofisopam to 10.14 for clobromazolam. In previous work, the highest predicted log 1/c value had been 8.88 for flunitrazolam [19]. In this work, four benzodiazepines had higher predicted binding affinities to the GABA_A_ receptor than flunitrazolam. They were fluclotizolam (8.91), difludiazepam (9.16), flualprazolam (10.13), and clobromazolam (10.14).

Previously, the greatest binding affinities had been reported for triazolobenzodiazepines. It is of no surprise that three of the designer benzodiazepines in this work with the greatest predicted binding affinities (fluclotizolam, flualprazolam, and clobromazolam) were also triazolobenzodiazepines. The exception was difludiazepam which had a predicted binding affinity of 9.16, but is a 1,4-benzodiazepine. The presence of halogenated groups at the ortho position of the phenyl ring, as with difludiazepam, is also thought to lead to enhanced activity at the GABA_A_ receptor [45].

Very little is known about these designer benzodiazepines. Some pharmacokinetic parameters have been reported for clobromazolam after a self-ingestion study [46]. An analysis of user reports has indicated that fluclotizolam is reported to be a potent benzodiazepine, with a dose of more than 0.75 mg reported as having a ‘heavy effect’ [47]. Flualprazolam has been reported in a number of DUID incidences, an ‘anaesthesia robbery’ case, as well as being detected post-mortem in a number of intoxications [48–51].

Tofisopam had the lowest predicted binding affinity of 5.03. Although classed a benzodiazepine, tofisopam is structurally different, because it is a 2,3-benzodazepine. Tofisopam is not thought to bind to the benzodiazepine site of the GABA_A_ receptor [52]. It is reported to exert its mechanism of action by inhibiting phosphodiesterase isoenzymes and only possesses anxiolytic properties in contrast to the sedative properties reported for other benzodiazepines [53].

Knowledge of the pharmacodynamics of designer benzodiazepines is also important to understand their potential effect and harms, and the use of QSAR provides an easy and inexpensive method of doing so. In this work, the binding affinities for the GABA_A_ receptor, expressed as log 1/c, were calculated from a QSAR model for various designer benzodiazepines. Most notably, four designer benzodiazepines that have appeared since 2017 (fluclotizolam, difludiazepam, flualprazolam, and clobromazolam) had predicted binding affinities to the GABA_A_ receptor that were greater than those previously reported [19]. Whether this was by chance or whether there is a concerted attempt to create benzodiazepines that exhibit a greater potency is unclear.

## Conclusions

30 designer benzodiazepines are currently monitored by the EMCDDA, with this number increasing every year. Knowledge of the blood-to-plasma ratios are required to fully interpret concentrations of benzodiazepines appearing as new psychoactive substances. QSAR modelling allows for a quick investigation of the pharmacology of designer benzodiazepines before biological data are likely to be available. Both the blood-to-plasma ratios and binding affinities exhibited a wide range of values highlighting the importance and necessity of accurate data to understand these new psychoactive substances.

## Supplementary Information

Below is the link to the electronic supplementary material.

Supplementary file1 (DOCX 75 KB)
