# Supplementary material for: The blood-to-plasma ratio and predicted GABAA-binding affinity of designer benzodiazepines
Source: Forensic Toxicol. 2022 Mar 16;40(2):349–56. doi: 10.1007/s11419-022-00616-y (PMC9715504; doi:10.1007/s11419-022-00616-y)
Supplement: Supplementary file 1 — Supplementary file1 (DOCX 75 KB) [file 11419_2022_616_MOESM1_ESM.docx]

# **Supplementary material**

**The blood to plasma ratio and predicted GABA_A_ binding affinity of designer benzodiazepines**

Kieran R Manchester^1*^, Laura Waters^2^, Shozeb Haider^3^, Peter D Maskell^4^

^1^School of Nursing and Healthcare Leadership, University of Bradford, Bradford. UK

^2^School of Applied Sciences, University of Huddersfield, Huddersfield. UK.

^3^School of Pharmacy, University College London, London. UK.

^4^Forensic Medicine and Science, University of Glasgow, Glasgow. UK

*Author for correspondence: K.R.Manchester@bradford.ac.uk

## **GC–MS validation methodology**

Five concentrations (10, 25, 50, 100, 250 µM) were used to assess linearity (three repetitions per concentration). A two-sample F-test was performed to analyse the concentration-response variation, and weighting factors (1/x or 1/x^2^) were used to obtain a linear fit [54, 55].

The LOQ was determined by analysis of the standard deviation of the response (σ) and the slope (S):

$LOQ=\frac{10\sigma}{S}$ (1)

The slope was determined from the calibration plot of the analyte. The standard deviation of the response was measured by analysing five blank samples and calculating the response at the retention time of each analyte. The LOD was calculated in a similar manner to the LOQ, from an analysis of the standard deviation of the response and the slope:

$LOD=\frac{3.3\sigma}{S}$ (2)

The slope and the standard deviation of blank samples at the retention time of the analyte was used in a similar manner to determination of the LOQ.

Interday and intraday accuracy were measured for three concentrations: 10, 50 and 250 µM (three repetitions per concentration). Interday accuracy was measured over three consecutive days.

Interday and intraday precision were measured for three concentrations 10, 50 and 250 µM (three repetitions per concentration). Interday precision was measured over three consecutive days.

## **GC–MS validation results**

### **Linearity**

### Linearity was lower than expected for some compounds in this study. The lowest coefficient of determination (R^2^) was 0.970 for etizolam and the highest was 0.992 for nitrazepam. However, all four compounds had calculated blood to plasma ratios that were within the literature ranges. This allowed for determination of the blood to plasma ratios for the designer benzodiazepines in this work.

### **Accuracy and Precision**

The highest interday accuracy was 100.3 % for pyrazolam (50 µM) and the lowest interday accuracy was 86.6% for meclonazepam (50 µM). The highest intraday accuracy was 101.2 % for nitrazepam (50 µM) and the lowest intraday accuracy was 110.6% for deschloroetizolam (250 µM). The lowest interday precision was 14.3 % for meclonazepam (50 µM) and the highest interday precision was 3.8 % for phenazepam (10 µM) and pyrazolam (10 µM). The lowest intraday precision was 11.0 % for etizolam (10 µM) and the highest intraday precision was 2.4 % for pyrazolam (250 µM). All calculated values for accuracy and precision were within ± 15 % which is similar to other GC–MS methods described in the literature [56].

**References**

[54] Gu H, Liu G, Wang J, Aubry A-F, Arnold ME (2014) Selecting the correct weighting factors for linear and quadratic calibration curves with least-squares regression algorithm in bioanalytical LC-MS/MS assays and impacts of using incorrect weighting factors on curve stability, data quality, and assay performance. Anal Chem 86:8959–8966. https://doi.org/10.1021/ac5018265

[55] Duer WC, Ogren PJ, Meetze A, Kitchen CJ, Von Lindern R, Yaworsky DC, Boden C, Gayer JA (2008) Comparison of ordinary, weighted, and generalized least-squares straight-line calibrations for LC-MS-MS, GC-MS, HPLC, GC, and enzymatic assay. J Anal Toxicol 32:329–338. https://doi.org/10.1093/jat/32.5.329

[56] Kollipara S, Bende G, Agarwal N, Varshney B, Paliwal J (2011) International guidelines for bioanalytical method validation: A comparison and discussion on current scenario. Chromatographia 73:201–217. https://doi.org/10.1007/s10337-010-1869-2

**Table S1** Compound retention time and target ions (quantification ion underlined), LOQ and LOD for GC–MS for all compounds in the study

| **Compound** | **Retention time (minutes)** | **Target ions**  **(*m*/*z*)** | **LOQ**  **(µM)** | **LOD**  **(µM)** |
| --- | --- | --- | --- | --- |
| Chlorpromazine | 9.66 | 58, 86, 272, 318 | 0.74 | 0.41 |
| Diazepam | 9.50 | 165, 221, 256, 283 | 0.60 | 0.27 |
| Nitrazepam | 13.20 | 206, 234, 253, 280 | 1.68 | 0.50 |
| Quinine | 10.76 | 96, 136, 215, 287 | 1.62 | 0.49 |
| Diclazepam | 13.71 | 77, 239, 279, 308 | 0.76 | 0.29 |
| Deschloroetizolam | 9.93 | 255, 283, 291, 318 | 1.33 | 0.45 |
| Etizolam | 14.40 | 239, 266, 313, 342 | 1.28 | 0.28 |
| Meclonazepam | 13.97 | 181, 222, 341, 370 | 1.09 | 0.27 |
| Phenazepam | 11.72 | 240, 286, 294, 328 | 0.75 | 0.21 |
| Pyrazolam | 9.80 | 75, 285, 321, 350 | 1.39 | 0.38 |

| **Compound** | **Coefficient of determination** | **Concentration (µM)** | | | | | | | | | | | |
| --- | --- | --- | --- | --- | --- | --- | --- | --- | --- | --- | --- | --- | --- |
|  |  | **250 (*n* = 3)** | | | | **50 (*n* = 3)** | | | | **10 (*n* = 3)** | | | |
|  |  | **Interday Precision RSD** | **Intraday Precision RSD** | **Interday Accuracy (%)** | **Intraday Accuracy (%)** | **Interday Precision RSD** | **Intraday Precision RSD** | **Interday Accuracy (%)** | **Intraday Accuracy (%)** | **Interday Precision RSD** | **Intraday Precision RSD** | **Interday Accuracy (%)** | **Intraday Accuracy (%)** |
| Chlorpromazine | 0.986 | 11.9 | 5.6 | 109.2 | 105.0 | 5.6 | 8.3 | 105.8 | 107.6 | 11.6 | 8.3 | 96.4 | 97.5 |
| Diazepam | 0.988 | 5.0 | 6.4 | 97.3 | 97.9 | 9.0 | 2.5 | 96.7 | 106.5 | 7.1 | 5.5 | 104.4 | 94.0 |
| Nitrazepam | 0.992 | 5.1 | 4.6 | 97.0 | 95.6 | 9.9 | 5.3 | 94.9 | 101.2 | 6.3 | 7.8 | 98.0 | 103.0 |
| Quinine | 0.973 | 12.7 | 3.7 | 107.0 | 108.7 | 10.3 | 5.3 | 106.9 | 95.2 | 9.7 | 4.4 | 103.4 | 104.4 |
| Diclazepam | 0.977 | 4.7 | 6.5 | 93.7 | 93.6 | 7.5 | 6.1 | 87.6 | 91.5 | 6.7 | 6.8 | 101.7 | 107.6 |
| Deschloroetizolam | 0.990 | 4.7 | 3.5 | 93.9 | 110.6 | 6.7 | 10.1 | 103.5 | 97.3 | 6.7 | 6.4 | 105.3 | 103.2 |
| Etizolam | 0.970 | 9.6 | 6.1 | 93.3 | 103.2 | 12.3 | 10.5 | 92.5 | 94.1 | 8.4 | 11.0 | 97.6 | 107.4 |
| Meclonazepam | 0.976 | 5.6 | 3.8 | 107.2 | 104.5 | 14.3 | 5.6 | 86.6 | 97.0 | 7.0 | 6.0 | 93.0 | 107.3 |
| Phenazepam | 0.991 | 3.9 | 6.2 | 104.9 | 106.0 | 7.0 | 8.4 | 87.2 | 96.9 | 3.8 | 6.7 | 90.0 | 95.2 |
| Pyrazolam | 0.990 | 8.6 | 2.4 | 100.9 | 98.1 | 6.4 | 8.1 | 100.3 | 104.3 | 3.8 | 4.7 | 108.9 | 103.5 |

**Table S2** Coefficients of determination, interday/intraday precision, and interday/intraday accuracy for GC–MS for all compounds in this study

| Name | Substitutions | | | | Log 1/c predicted | Basic structure |
| --- | --- | --- | --- | --- | --- | --- |
|  | R7 | R1 | R2' | R3 |  |  |
| Cinazepam | Br | - | Cl | OCHO(CH_2_)_2_COOH | 7.11038 |  |
| Gidazepam | Br | CH_2_CONHNH_2_ | - | - | 8.3262 |  |
| Desalkylgidazepam | Br | - | - | - | 7.97322 |  |
| Difludiazepam^a^ | Cl | CH_3_ | F | - | 9.16362 |  |
| Norfludiazepam | Cl | - | F | - | 8.85335 |  |
| Thionordazepam^b^ | Cl | - | - | - | 7.08873 |  |
| ^a^Difludiazepam also has a fluorine group substituted at the R_6’_ position.  ^b^Thionordazepam has a thione group at R2 instead of a ketone group. | | | | | | |
|  | | | | | |  |

**Table S3** Predicted binding affinities (log 1/c) and structures for designer benzodiazepines sharing the 1,4-benzodiazepine structure

| Name | Substitutions | | | Log 1/c predicted | Basic structure |
| --- | --- | --- | --- | --- | --- |
|  | **R_8_** | **R_1_** | **R_2'_** |  |  |
| Clobromazolam | Br | CH_3_ | Cl | 10.14 |  |
| Flualprazolam | Cl | CH_3_ | F | 10.1289 |  |

**Table S4** Predicted binding affinities (log 1/c) and structures for designer benzodiazepines sharing the triazolobenzodiazepine structure

**Table S5** Predicted binding affinities (log 1/c) and structures for designer benzodiazepines sharing the thienotriazolodiazepine structure

| Name | Substitutions | | | Log 1/c predicted | Basic structure |
| --- | --- | --- | --- | --- | --- |
|  | R_9_ | R_2_ | R_2'_ |  |  |
| Fluclotizolam | CH_3_ | Cl | F | 8.90603 |  |

| Name | Log 1/c predicted | Structure |
| --- | --- | --- |
| Bentazepam | 6.8769 |  |

**Table S6** Predicted binding affinity (log 1/c) and structure for bentazepam (thienodiazepine)

**Table S7** Predicted binding affinity (log 1/c) and structure for tofisopam (2,3-benzodiazepine)

| Name | Log 1/c predicted | Structure |
| --- | --- | --- |
| Tofisopam | 5.02924 |  |
